# PD186 Treat-To-Target In Gout: Are Private General Practitioners In Malaysia Riding The Evidence-Based Wave?

**DOI:** 10.1017/S0266462324004082

**Published:** 2025-01-07

**Authors:** Siti Mariam binti Mohtar, Maslinor Ismail, ‘Aziezah Norul Anhar, Izzuna Mudla Mohamed Ghazali

## Abstract

**Introduction:**

Gout is an inflammatory disease that can cause severe pain and permanent joint destruction if it is not treated appropriately. Despite readily available treatments, the management of gout in primary care is suboptimal. Hence, this study aimed to investigate gout knowledge and compliance with evidence-based clinical practice guidelines (CPGs) among private general practitioners (GPs) in Malaysia.

**Methods:**

A cross-sectional study using stratified random sampling was conducted among private GPs in Selangor, Putrajaya, and Kuala Lumpur from October to December 2023. The survey consisted of four parts: inclusion and exclusion criteria, sociodemographic data, knowledge, and practice domain. A total of 15 questions were posed in the knowledge domain and nine questions in the practice domain. Statistical analysis was performed using R software (version 4.3.2). Descriptive statistics were used for initial analyses. Proportions were calculated for categorical data, which were tested using a chi-square test. A p-value of less than 0.05 was considered statistically significant.

**Results:**

The preliminary results from 203 private GPs showed that 64 percent had moderate knowledge of the management of gout. About two-thirds of the GPs were aware of local evidence-based CPGs on gout, but only 20 percent had good knowledge of gout. In the practice domain, only 65.5 percent of private GPs were aiming for a serum urate level of less than six milligrams per deciliter when starting urate-lowering therapy for gout.

**Conclusions:**

Although most private GPs had moderate or good knowledge of gout, clinical practice was not fully concordant with the recommendations of local evidence-based CPGs. Our findings provide a foundation for local CPG developers and professional societies to enhance their strategies for implementing evidence-based CPGs and encouraging GPs in Malaysia to incorporate evidence-based practices into their clinical decisions.

